# Cellular reprogramming is driven by widespread rewiring of promoter-enhancer interactions

**DOI:** 10.1186/s12915-023-01766-0

**Published:** 2023-11-20

**Authors:** Miao Wang, Bing He, Yueling Hao, Divyaa Srinivasan, Jatin Shrinet, Peter Fraser

**Affiliations:** Department of Biological Science, Florida State University, Tallahassee, FL USA

**Keywords:** Pre-B cell, Macrophage, C/EBPα, Hi-C, Promoter-Capture Hi-C(PCHi-C), Long-range interactions

## Abstract

**Background:**

Long-range interactions between promoters and *cis*-regulatory elements, such as enhancers, play critical roles in gene regulation. However, the role of three-dimensional (3D) chromatin structure in orchestrating changes in transcriptional regulation during direct cell reprogramming is not fully understood.

**Results:**

Here, we performed integrated analyses of chromosomal architecture, epigenetics, and gene expression using Hi-C, promoter Capture Hi-C (PCHi-C), ChIP-seq, and RNA-seq during trans-differentiation of Pre-B cells into macrophages with a β-estradiol inducible C/EBPαER transgene. Within 1h of β-estradiol induction, C/EBPα translocated from the cytoplasm to the nucleus, binding to thousands of promoters and putative regulatory elements, resulting in the downregulation of Pre-B cell-specific genes and induction of macrophage-specific genes. Hi-C results were remarkably consistent throughout trans-differentiation, revealing only a small number of TAD boundary location changes, and A/B compartment switches despite significant changes in the expression of thousands of genes. PCHi-C revealed widespread changes in promoter-anchored loops with decreased interactions in parallel with decreased gene expression, and new and increased promoter-anchored interactions in parallel with increased expression of macrophage-specific genes.

**Conclusions:**

Overall, our data demonstrate that C/EBPα-induced trans-differentiation involves few changes in genome architecture at the level of TADs and A/B compartments, in contrast with widespread reorganization of thousands of promoter-anchored loops in association with changes in gene expression and cell identity.

**Supplementary Information:**

The online version contains supplementary material available at 10.1186/s12915-023-01766-0.

## Background

*Cis*-regulatory elements such as enhancers and promoters of genes they control can be separated by large genomic distances [1]. However, during gene transcription, they are found in very close physical proximity [2]. Gene expression is regulated by temporal-spatial, enhancer-promoter interactions. Such long-range interactions often bypass proximal genes to exert control over specific distal target genes [1, 3]. Deciphering the principles and mechanisms underlying enhancer-promoter dynamics is essential for understanding the molecular mechanism of gene control in cell differentiation and development.

Genome-wide contact frequencies determined by Hi-C have revealed higher-order chromatin structural features. Topologically associated domains (TADs) are large megabase-size genomic regions of increased self-interaction that are partially insulated from contacts with neighboring domains [4, 5]. Various studies have reported conflicting results on TAD organization in response to environmental stimuli. Some studies show that TADs are largely invariant among different cell types and in response to environmental signals [4, 6, 7], while others have reported significant TAD re-organization in response to stimuli and between cell types [8–10]. Further studies have shown that transcription factors drive dynamic change of chromatin interactions between regulatory elements and genes within TADs during ESC differentiation, human fibroblast trans-differentiation, and B cell reprogramming [11–13].

Analysis of Hi-C heatmaps has revealed a “plaid pattern” of increased or decreased contact frequencies between domains which corresponds with their activity state. Active domains tend to contact other active domains, while inactive domains tend to contact other inactive domains, indicating that the nuclear genome is organized into two nuclear compartments referred to as A (active compartment) and B (inactive compartment) [14]. Here again, several studies have investigated compartment changes during cell differentiation in both mouse and human cell types and reported conflicting results. For example, human embryonic stem cell (ESC) differentiation to four different lineages showed substantial switching between A and B compartments [6] while during cell differentiation and development, studies show there is minimal A–B switching [15–18].

Hi-C has been instrumental in discovering and defining the principles of the above aspects of higher-order genome organization. However in the absence of billions of read-pairs, Hi-C lacks the resolution to identify significant interactions between individual promoters and their long-range regulatory elements. Promoter Capture Hi-C (PCHi-C) identifies significant interactions between individual promoters and their long-range regulatory elements in an unbiased manner [19–21]. PCHi-C studies have shown dynamic rewiring of enhancer-promoter interactions during human ESC differentiation to neuroectodermal cells [22], during mouse adipocyte differentiation [7], and during keratinogenesis [23] suggesting that promoter interaction changes regulate gene expression associated with cell-fate transitions. These studies are in agreement with Highly Integrative Chromatin Immunoprecipitation (HiChIP) experiments, which show chromatin reorganization during cell trans-differentiation and reprogramming [24–26]. However, the degree to which promoter interactions are reorganized during direct lineage conversion remains poorly understood.

Pre-B cells are precursors of B lymphocytes, which are adaptive immune cells of the lymphoid lineage [27, 28]. Macrophages are phagocytic cells of the myeloid lineage and are responsible for detecting, engulfing, and destroying pathogens [29]. The lymphoid and myeloid lineages exhibit distinct gene expression patterns maintained by lineage-restricted transcription factors [30–32]. The CCAAT enhancer binding protein alpha (C/EBPα) is essential for the development of the myeloid lineage [33]. An inducible Pre-B cell line (c10) with a C/EBPαER transgene has been well studied for its ability to reproducibly trans-differentiate into macrophages [34–36]. C/EBPα has previously been shown to bind to myeloid enhancers in Pre-B cells and regulate myeloid cell type-specific genes [33].

In this study, we used ChIP-seq, RNA-seq, Hi-C, and PCHi-C data to investigate higher-order chromatin organization and promoter interaction dynamics associated with gene expression changes during C/EBPαER-induced trans-differentiation of mouse Pre-B cells into macrophages over a 48-h period [34, 37]. Our findings show that extensive temporal rewiring of promoter-anchored interactions during the switch from a Pre-B cell-specific to a macrophage-specific transcription program constitutes the major alterations in genome organization during trans-differentiation.

## Results

### Transcriptional dynamics during rapid conversion of Pre-B cells into macrophages

**Fig. 1:**
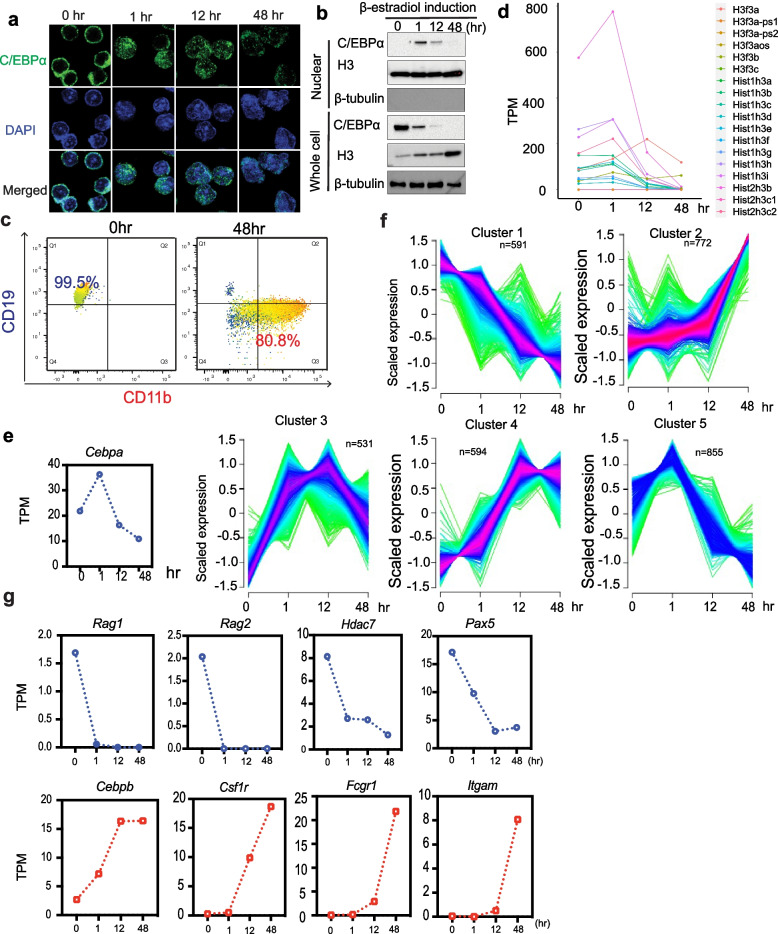
Timescale conversion of Pre-B cells into Macrophages. **a** Immunofluorescence staining of C/EBPα showing cellular localization of C/EBPα proteins at different time points. **b** Western blot analysis showing C/EBPα protein level in nuclear and whole cell extracts upon β-estradiol induction. **c** FACS analysis of the percentage of cells that express Pre-B cell-specific surface marker, CD19, and macrophage-specific cell surface marker, CD11b. **d** Gene expression level (transcripts per million) of genes encoding histone3. **e** Gene expression level (transcripts per million) of Cebpa gene. **f** Fuzzy c-means clusters of differentially expressed genes from 0h, 1h, 12h, and 48h during trans-differentiation. **g** Gene expression level (transcripts per million) of Pre-B cell-specific genes (*Rag1*, *Rag2*, *Hdac7*, *Pax5*) (blue), and macrophage-specific genes (*Cebpb*, *Csf1r*, *Fcgr1*, *Itgam*) (red) during Pre-B trans-differentiation

To study the role of promoter-enhancer interactions during Pre-B cell trans-differentiation into macrophages, we employed direct lineage conversion of the C10 mouse Pre-B cell line which harbors an estradiol-inducible rat C/EBPα fused to the estrogen hormone receptor binding domain (C/EBPαER) [34, 38]. Upon addition of β-estradiol, C10 cells are converted into macrophages within 48 h. Immunofluorescence staining and western blot analysis show that C/EBPα protein is rapidly translocated from the cytoplasm to the nucleus within 1 h of β-estradiol induction and gradually decreases over 48 h (Fig. 1a, b). We verified trans-differentiation efficiency by FACS to determine the percentage of cells expressing the Pre-B cell-specific cell surface marker CD19 and macrophage-specific cell surface marker CD11b. Over 48 h of induction, the cells went from 99.5% CD19 positive to greater than 80% CD11b positive (Fig. 1c), demonstrating efficient differentiation into macrophages.

Surprisingly, we found that Histone 3 (H3) levels increased in the whole cell extracts through Pre-B trans-differentiation (Fig. 1b). To analyze gene expression dynamics during trans-differentiation, we prepared triplicate RNA-seq libraries from each of the four time points (0 h, 1 h, 12 h, and 48 h). Principal component analysis (PCA) shows three biological replicates cluster together, indicating high similarity and library quality (Additional file 1: Fig. S1a). Allele-specific analysis of H3 gene expression shows that the increased H3 protein level is due to expression of the histone variant H3.3 (*H3f3a* and *H3f3b*) genes (Fig. 1d). H3.3 is often referred to as the replacement histone as it is incorporated into transcribed chromatin throughout the cell cycle [39, 40]. Expression of canonical H3 alleles decreased dramatically after induction with CEBP/α (Fig. 1d), consistent with previous studies showing that CEBP/α arrests cell proliferation [41–43]. Increased levels of H3.3 may be required for the large-scale changes in gene expression that occur upon trans-differentiation. RNA-seq analysis also shows that expression of the Rat C/EBPα transgene initially increases at 1h and then decreases by 48h (Fig. 1e). The endogenous C/EBPα gene (mouse specific) was not expressed throughout the time course (Additional file 1: Fig. S1b). We identified thousands of differentially expressed genes (DEGs) comparing RNA seq data from 1h, 12h, and 48h to 0h (Additional file 1: Fig. S1c) and clustered them to reveal 5 clusters of genes with different expression profiles (Fig. 1f, Additional file 2: Table S1). Cluster 1 genes decrease in expression from 0 to 48h. Gene ontology (GO) indicates the enrichment of genes involved in B-cell-related functions: B cell activation, lymphocyte activation, and leukocyte activation (Additional file 1: Fig. S1d). A few examples of these genes are *Rag1*, *Rag2*, *Hdac7*, and *Pax5* (Fig. 1g, Additional file 1: Fig. S1e). Cluster 2 genes increase in expression from 0h to 48h, and GO analysis shows enrichment of genes involved in the cellular response to molecule of bacterial origin, cellular response to biotic stimulus, and other macrophage-related functions (Additional file 1: Fig. S1d); some examples are *Cebpb*, *Csf1r*, *Fcgr1*, and *Itgam* (Fig. 1g and Fig.S1e). Similar to other studies, we observed downregulation of B lineage transmembrane protein gene *Cd19* and B cell receptor complex signaling genes *Blnk, Cd79a*, *Cd79b*, *Vpreb1*, Vpreb2, and *Vpreb3*, while myeloid marker genes such as granulocyte collagenase 8 *(Mmp8),* macrophage scavenger receptor (*Msr1*), myeloid restricted serine protease C (*Ctsc*), and the myeloid cytokine-dependent chemokine 6 (*Ccl6*) were upregulated, (Additional file 1: Fig. S1f) [35, 44]. Together, these results show that nuclear translocation of C/EBPαER results in rapid diminution of the Pre-B cell-specific gene expression program and progressive establishment of macrophage-specific gene expression, indicating efficient conversion of Pre-B cells to macrophages.

### Higher-order chromatin organization during Pre-B cell trans-differentiation

**Fig. 2:**
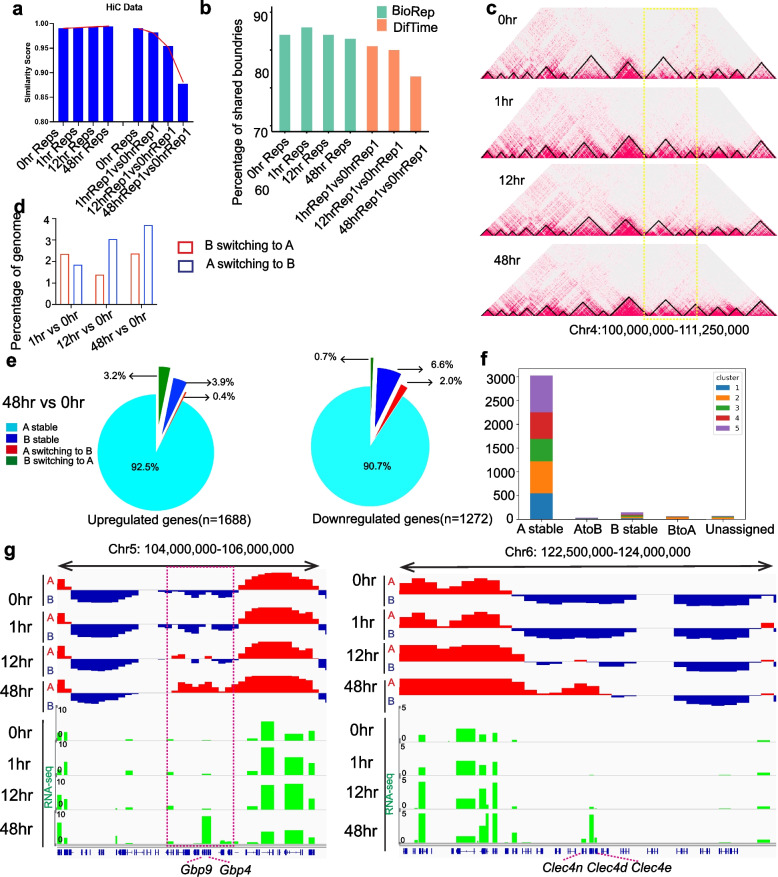
Higher-order chromatin organization during Pre-B cell trans-differentiation. **a** Similarity scores between the replicates and different time points. Stratum adjusted correlation coefficient (SCC method) was used to quantify the similarity score by comparing HiC interaction matrix at 500 kb resolution. Reps, replicates. **b** Percentage of shared TAD boundaries between biological replicates and among samples from different time points. BioRep, comparison between biological replicates at each time point. DifTime, comparison between samples from different time points. **c** TAD structure at a region on chromosome 4—black triangles represent TADs, and yellow lines demarcate regions showing differences in TAD boundaries between time points. **d** Percentage of the genome that switches compartment (either A-to-B or B-to-A) during trans-differentiation. **e** The percentage of compartment change for significantly upregulated and downregulated genes (48h vs 0h). **f** The number of DEGs in different Fuzzy c-means clusters grouped by various compartments change. Unassigned: genes are not able to call compartments. **g** Examples of genes that are in B compartment but switch to A compartment. Gene expression tracks in green

To investigate higher-order chromatin dynamics during trans-differentiation, we performed Hi-C in duplicate at the same time points. HiCUP analyses reveal > 70% of di-tags are *cis*-contacts greater than 10Kb, approximately 15% *cis*-contacts < 10 Kb, and ~ 14% *trans* contacts, indicating very high-quality Hi-C libraries (Additional file 1: Fig. S2a) [45]. Comparison of Hi-C matrices at 500-kb resolution showed very high similarity scores between replicates at all time points, and progressively lower similarity when comparing uninduced cells to trans-differentiation time points (Fig. 2a). Hi-C heatmaps at 100-kb and 25-kb resolution did not show gross changes in overall architecture between different time points (Additional file 1: Fig. S2b). We found approximately 3000 TADs at each time point, ranging in size from a few hundred kilobases to a few Mbs, with an average size of approximately 800kb (Additional file 1: Fig. S3a). TAD boundaries show higher reproducibility between biological replicates than between different time points (Fig. 2b), indicating a small percentage of boundaries change during trans-differentiation (Fig. 2b, c). We observed that 17,762 genes (32.08% of total genes) are located within TADs that show boundary changes (48h vs 0h). Of these genes, 937 are differentially expressed genes (DEGs) (31.36% of total DEGs). Fisher’s exact test shows that genes within TADs that show boundary changes are not enriched for DEGs (*p* = 0.40). GO analysis of coding genes (*n* = 6937) within TADs that show boundary changes shows enrichment for immune processes, such as response to chemokine, response to dsRNA, and response to pheromone (Additional file 1: Fig. S3b). These results show that a majority of TAD boundaries are conserved, consistent with previous reports of TAD conservation across different cell types [4, 6, 7]. However, we find that some boundaries do change and are associated with changes in gene expression within the flanking TADs suggesting that alteration of TAD boundaries is involved, though not a major feature driving the observed widespread changes in gene expression.

We called A/B compartments using principal component analysis (PCA). PC1 value of PCA shows a higher correlation between biological replicates than between different time points (Additional file 1: Fig. S3c). We found as expected A compartment genes have significantly higher expression levels than those in B compartments (Additional file 1: Fig. S3d). Previous reports indicate that although most compartments are stable across different cell types, some compartments do switch status during trans-differentiation in a cell-type-specific manner, reflecting cell-type-specific changes in transcription [6, 11]. We observed that a small fraction of the genome switches compartment status (either A-to-B or B-to-A) during trans-differentiation (Fig. 2d). Looking at the relationship between gene expression changes and compartment switching, we observed that of the 1688 upregulated genes from 0h to 48h, only 54 (3.2%) switched from the B compartment to the A compartment, and only 25 (2%) of 1272 downregulated genes switched from A compartment to B compartment (Fig. 2e). Greater than 90% of DEGs (either up- or downregulated) remained in stable A compartments throughout trans-differentiation (Fig. 2e and Additional file 1: Fig. S3e). Correlation between the A/B compartment switches and transcriptome clusters shows as expected A to B switches are enriched in cluster 1 which contains downregulated genes, and B to A switches are enriched in clusters 2, 3, and 4 which are composed of upregulated genes (Fig. 2f). The expression changes of genes from B to A compartments are significantly greater than those maintained in the same compartments, while the expression changes of genes from A to B compartments are notably lower (Additional file 1: Fig. S3f). GO analysis indicates that genes that switch compartments from A to B are involved in immunoglobulin production and genes that switch compartments from B to A are associated with Pre-B differentiation and macrophage-related functions, such as antifungal immune response, stimulatory C-type lectin receptor signaling pathway, and cellular response to type II interferon (Additional file 1: Fig. S3g). For example, *Gbp* family genes, G*bp9* and *Gbp4*, that are involved in response to interferon-gamma show increased expression at 48h accompanied by compartment B to A switch (Fig. 2g) [46]. Similarly, *Clec4* family genes, *Clec4n*, *Clec4d*, and *Clec4e*, involved in macrophage defense response to other organisms also show increased expression along with the B to A compartment switch (Fig. 2g) [47]. In summary, trans-differentiation involves a limited number of changes in TAD boundaries and A/B compartments.

### Promoter-anchored chromatin interactions during trans-differentiation

**Fig. 3:**
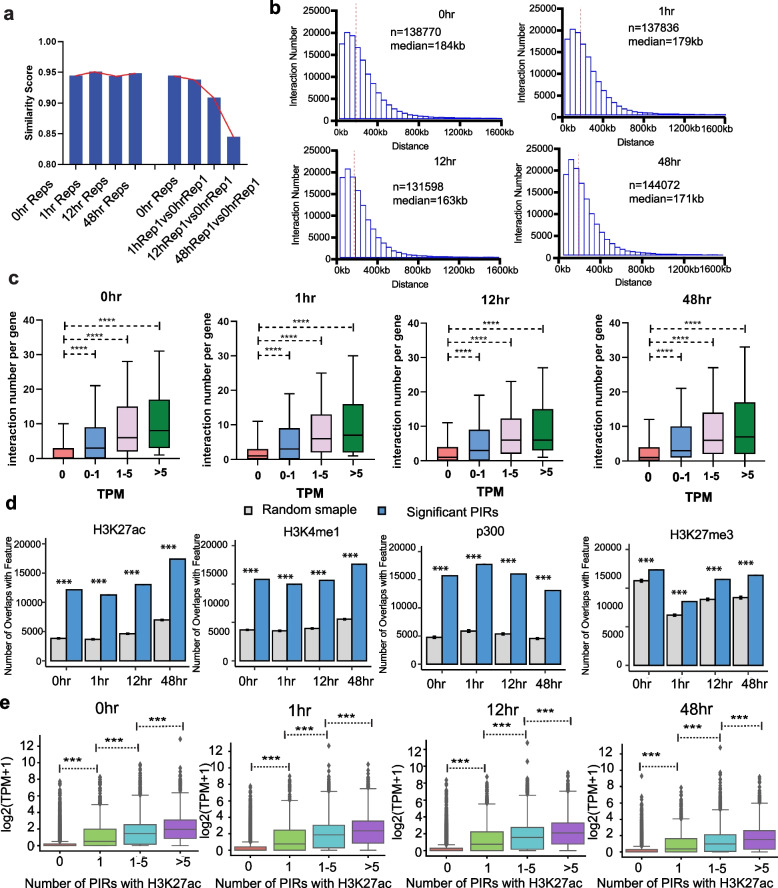
Integrative analysis of promoter-anchored chromatin interactions, transcriptome, and epigenetics during Pre-B cell trans-differentiation. **a** Similarity score between replicates and different time points of PCHi-C interaction matrix at 500-kb resolution was quantified using stratum-adjusted correlation coefficient (SCC) method. **b** Distribution of distances between promoter and promoter interacting regions (PIRs) at 0h, 1h, 12h, and 48h time points. Red dashed line indicates the median distance of significant interactions. **c** Number of interactions per gene classified based on different expression levels (TPM = 0 (n1), TPM = 0–1 (n2), TPM = 1–5 (n3), TPM > 5 (n4)) at 0 h (n1 = 9601, n2 = 4669, n3 = 4116, n4 = 2202), 1h (n1 = 9025, n2 = 4588, n3 = 3772, n4 = 3203), 12h (n1 = 9184, n2 = 4721, n3 = 3970, n4 = 2713), and 48h (n1 = 9266, n2 = 5837, n3 = 3828, n4 = 1657) time points. An unpaired two-sided *t*-test is performed for the significance test. **d** Overlap of H3K27ac, H3K4me1, H3K27me3, and p300 peaks with significant versus randomly shuffled PIRs for cells at different time points. The gray bar represents the number of overlaps across 100 sets of randomly shuffled distance-matched PIRs (control) with 95% confidence interval for the means plotted. The chi-squared test is performed for the significance test. **e** The box plot displays gene expression values (log2(TPM + 1)) of promoters categorized into four groups based on the number of promoter-interacting regions (PIRs) marked by H3K27ac at four different time points. The groups are defined as follows: Group 0: Promoters without any PIRs marked by H3K27ac. Group 1: Promoters with only one PIR marked by H3K27ac. Groups 1–5: Promoters with one to five PIRs marked by H3K27ac. Group > 5: Promoters with more than five PIRs marked by H3K27ac. An unpaired two-sided *t*-test is performed for the significance test. All the box plot represents 25 and 75 percentiles with the median

To investigate promoter-anchored chromatin interactions during Pre-B cell trans-differentiation, we generated Promoter Capture Hi-C (PCHi-C) libraries at each time point. PCHi-C data quality was checked using HiCUP analysis (Additional file 1: Fig S4a). Comparison of the datasets revealed high similarity scores between replicates at all time points and progressively lower similarity scores as trans-differentiation advanced (Fig. 3a). Using CHICAGO (cut-off score = 5), we identified around 130,000 significant promoter interacting regions (PIRs) at each time point (Fig. 3b). Approximately 17% are promoter to promoter (bait to bait), and 83.4% are promoter to other genomic fragments, (Additional file 1: Fig. S4b). Promoter-anchored interactions across all time points show similar distributions over linear genomic distance (Fig. 3b) with median distances of 163 ~ 184kb.

*Cis*-regulatory elements, such as enhancers, far outnumber genes in the mammalian genome [48, 49]. In many cases, a gene will be contacted by multiple enhancers, which can be located at great genomic distances [1, 50]. We examined the relationship between the number of significant interactions per gene promoter and gene expression level. We found that genes possessing a larger number of interactions tend to have significantly higher gene expression (Fig. 3c) in agreement with previous studies [50–52].

Functionally active *cis*-regulatory elements are often enriched in specific histone modifications [53]. H3K27ac, H3K4me1, and p300 are often associated with active enhancers, whereas H3K27me3 modifications are generally associated with *Polycomb* repressed chromatin, bivalent domains, and poised enhancers [54–56]. To characterize PIRs during Pre-B cell trans-differentiation, we analyzed publicly available ChIP-seq data for H3K27ac, H3K4me1, H3K27me3, and p300 peaks [37] at PIRs compared to distance-matched, random control regions at all four time points. We found that PIRs are significantly enriched for active enhancer marks H3K27Ac, H3K4Me1, and p300 (Fig. 3d), consistent with active transcription of the contacted promoters. We also found that PIRs are significantly enriched with H3K27me3 peaks (Fig. 3d). To investigate the role of PIRs enriched in H3K27ac and H3K27me3 in gene expression, we classified promoters into four groups: Those with at least one PIR marked by H3K27ac, at least one PIR marked by H3K27me3, both, or no marks. Promoters contacted by PIRs with H3K27ac show significantly higher gene expression than the other three groups. Promoters contacted by PIRs with H3K27me3 show significantly lower gene expression than the other three groups (Additional file 1: Fig. S4c). We classified promoters into four groups based on the number of their PIRs marked by H3K27ac and compared their expression level. The results show that promoters connected to more than one PIR marked by H3K27ac peaks have significantly higher gene expression indicating a dose-dependent effect on transcription (Fig. 3e). To quantify the effect of H3K27me3, we focused on promoters contacting PIRs marked by H3K27me3 and not marked by H3K27ac. These promoters were then categorized into four groups based on the number of PIRs marked by H3K27me3. The results show that promoters that connected more than one PIR marked by H3K27me3 have a modest but significantly lower gene expression level than promoters that only have one PIR marked by H3K27me3, suggesting a dose-dependent effect of repressor-promoter interactions on gene expression (Additional file 1: Fig. S4d). These results demonstrate that active and repressive histone modifications at PIRs are associated with active and repressed transcription (respectively) of the promoters they contact, as expected.

### Dynamic rewiring of interactions during trans-differentiation

**Fig. 4:**
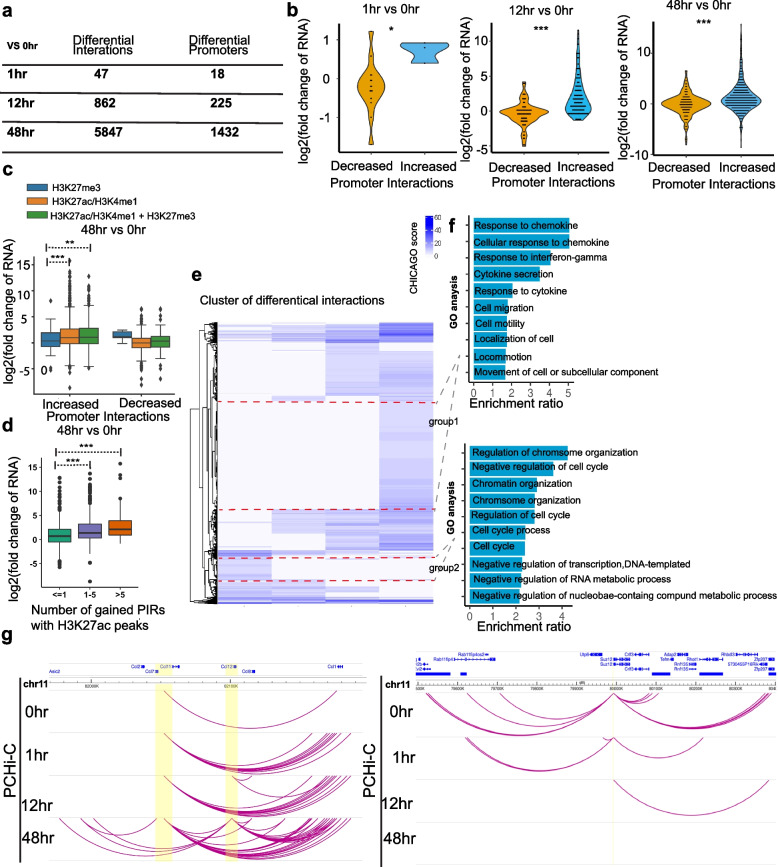
Dynamic rewiring of promoter-anchored chromatin interactions during trans-differentiation of Pre-B cells to macrophage. **a** Table shows the number of significant differential interactions when 1h, 12h, and 48h are compared to 0h time point. **b** Violin plots showing log2 fold changed expression of promoters based on alteration of their contact frequency (decreased and increased) when 1h (decreased = 15, increased = 3), 12 h (decreased = 77, increased = 143), and 48h (decreased = 313, increased = 1011) are compared to 0h time points. A two-sided Mann–Whitney *U* test is performed for the significance test. c The log2 fold change of gene expression on gene promoters significantly increased and decreased chromatin interactions (48h vs 0h) with histone modification marks. H3K27me3: promoter interacting regions (PIRs) overlap with at least one H3K27me3 peak, but do not overlap with either H3K27ac or H3K4me1. H3K27ac /H3K4me1: promoter interacting regions (PIRs) overlap with at least one either H3K27ac or H3K4me1, but do not overlap with H3K27me3. H3K27ac/H3K4me1 + H3K27me3: promoter interacting regions (PIRs) overlap with at least one either H3K27ac or H3K4me1 and overlap with H3K27me3. Increased: H3K27me3 (*n* = 74); H3K27ac/H3K4me1 (*n* = 668); H3K27ac/H3K4me1 + H3K27me3: (*n* = 299); decreased: H3K27me3 (*n* = 5); H3K27ac/H3K4me1 (*n* = 269); H3K27ac/H3K4me1 + H3K27me3 (*n* = 66). The unpaired two-sided *t*-test was performed to test significant differences between groups. The unpaired two-sided *t*-test was performed to test significant differences between groups. d Log2 fold change of gene expression based on the number of H3K27ac peaks on the increased PIRs (48h vs 0h). The number of histone mark peaks is classified into three groups. ≤ 1: Genes that gain chromatin interactions with less than one histone mark peaks (*n* = 469). 1–5: Genes that gain chromatin interactions with one to five histone mark peaks (*n* = 229). > 5: Genes that gain chromatin interactions with greater than five histone mark peaks (*n* = 44). The unpaired two-sided *t*-test was performed to test significant differences between groups. e Heatmap shows 5640 significantly different interactions clustered based on the Euclidean distance for interaction score (CHICAGO score greater than 5 in at least one of the groups). Group 1 is 48-h specific interactions (*n* = 2169). Group 2 is 0-h specific interactions (*n* = 395). f Enriched GO term for genes in Group 1 and Group 2. Top panel—GO terms for genes (*n* = 1190) involved in 48h specific interactions; lower panel—GO term for genes (*n* = 656) involved in 0h specific interactions. g Examples of significantly increased interactions at *Ccl7 *and *Ccl12 *loci (left panel) and significantly decreased interactions at *Suz12* loci (right panel) across time points

We measured significant differences in promoter interactions during trans-differentiation using ChicDiff [57] and found that the number of differential interactions increased dramatically during trans-differentiation. Compared to uninduced cells, we found 47 differential interactions involving 18 promoters at 1h, 862 differential interactions at 225 promoters at 12h, and 5847 differential interactions at 1432 promoters at 48h (Fig. 4a), Additional file 3: Table S2). To investigate the potential effect of differential interactions on gene expression, we classified promoters into two groups based on contact frequency (read-pair depth): increased (gain of contacts at 1h, 12h, or 48h compared to 0h) and decreased (decreased contact frequency between 1h, 12h, and 48h compared to 0h). These data show that contact frequency is positively correlated with gene expression. Promoters that gained contacts show significantly upregulated gene expression while promoters that lost contacts show significantly downregulated gene expression (Fig. 4b). To investigate the role of histone modifications on gene expression by chromatin looping, we classified the PIRs of significant differential promoter interactions into three categories based on overlap with different histone marks and quantified their target gene expression change. We found gene promoters that gained chromatin interactions with enhancer marks (either H3K27ac or H3K4me1) show significantly upregulated gene expression compared to gene promoters that only gain contact with repressor marks (H3K27me3) (Fig. 4c). Gene promoters that gained contact with repressor marks (H3K27me3) such as *Maf1* and *Sspb1* show downregulated gene expression (Additional file 1: Fig. S5a, Additional file 1: Fig. S5b). We also investigated the additive effect of gaining new enhancer contacts on gene expression changes by quantifying the number of gained PIRs with H3K27ac or H3K4me1 peaks and evaluated the corresponding gene expression changes at 48h compared to 0h. Promoters gaining interactions with multiple PIRs with enhancer marks exhibited significantly higher gene expression changes compared to those with interactions involving only one enhancer mark (Fig. 4d), Additional file 1: Fig. S5c). Taken together, these results suggest that gene expression changes during trans-differentiation are driven in part through widespread changes in promoter interactions with functional regulatory elements.

We clustered differential interactions to further investigate the dynamic rewiring of promoter interactions (Fig. 4e), Additional file 3: Table S2). The most dramatic change in differential interactions occurs between 12h and 48h. Gene ontology (GO) enrichment analysis shows that these genes are associated with macrophage-related functions, including response to chemokine, interferon-gamma, and cytokine, as well as cell migration and motility (Fig. 4f). For example, *Ccl7* and *Ccl12* gain interactions at 48h and have macrophage-related functions (Fig. 4g) [58, 59]. Interestingly, GO shows genes that rapidly lose interactions during trans-differentiation are involved in regulation of chromosome organization, cell cycle, and negative regulation of transcription, suggesting alterations in chromosome organization and cell cycle changes occur along with changes in gene expression (Fig. 4f). *Suz12* is an example of a gene that progressively loses interactions after induction (Fig. 4g). We identified gene promoters that lost interactions at 48h, and GO analysis shows enrichment of genes involved in type I interferon production and B cell activation (Additional file 1: Fig. S6a). Some examples of Pre-B cell-specific genes are *Pax5* and *Clcf1* (Additional file 1: Fig. S6b). These results indicate that gained and lost promoter-anchored interactions are a major contributor to cell fate transition.

### C/EBPα binds to interacting regions and modulates gene expression during Pre-B cell trans-differentiation

**Fig. 5:**
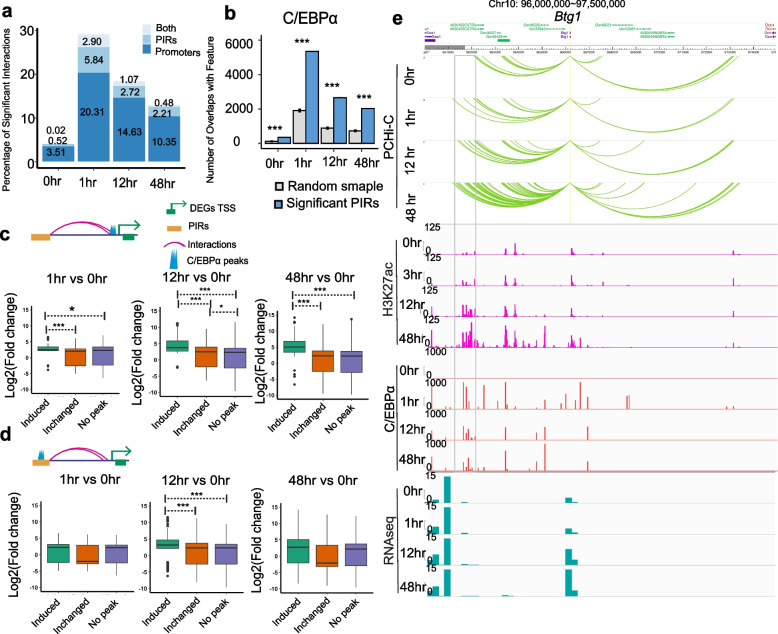
C/EBPα binds to promoters and PIRs and modulates gene expression during Pre-B cell trans-differentiation. **a** Percentage of significant interactions that overlap with C/EBPα binding peaks. Both: Percentage of significant interactions bound by C/EBPα at both promoter and PIRs anchors; PIRs: Percentage of significant interactions bound by C/EBPα only at PIRs anchors; Promoters: Percentage of significant interactions bound by C/EBPα only at Promoter anchors. **b** Overlap of C/EBPα peaks with significant PIRs versus randomly shuffled distance-matched fragments (control) for cells at different time points. The gray bar represents the number of overlaps across 100 sets of randomly shuffled distance-matched PIRs (control) with 95% confidence interval for the means plotted. The chi-squared test is performed for the significance test. **c**, **d** Box and whisker plots show DEGs expression between 0h and other time points based on differential C/EBPα binding at (**c**) promoters or (**d**) promoter interaction regions. *Induced*: Additional time specific C/EBPα binding at promoters or PIRs. *Unchange:* Stable C/EBPα binding at promoters or PIRs. *No peak:* no C/EBPα binding at promoters or promoter interaction regions. Promoter: 1h vs 0h, Induced (*n* = 52), Unchanged (*n* = 70), No peaks (*n* = 283). 12h vs 0h, Induced (*n* = 89), Unchanged (*n* = 172), No peak (*n* = 1168). 48h vs 0h, Induced (*n* = 94), Unchanged (*n* = 286), No peak (*n* = 2592). PIRs: 1h vs 0h, Induced (*n* = 76), Unchanged (*n* = 72), No peaks (*n* = 77). 12h vs 0h, Induced (*n* = 152), Unchanged (*n* = 262), No peak (*n* = 428). 48h vs 0h, Induced (*n* = 251), Unchanged (*n* = 427), No peak (*n* = 1067). The box plot represents 25 and 75 percentiles with the median. A two-sided Mann–Whitney *U* test is performed for the significance test. **e** *Btg1* region that shows promoter interaction changes accompanied by H3K27ac (pink) and C/EBPα ChiP-seq peaks (red) at interacting regions, along with gene expression (green). The gray box shows gained PIRs of the Btg1 promoter with increased C/EBPα binding and gain of H3K27ac

The C/EBPα transcription factor is a master regulator of macrophage identity [33]. We investigated how C/EBPα binding to interacting regions affects transcriptional changes. We identified 23,434 C/EBPα binding peaks 1h after induction, and a gradual decrease at 12h and 48h (Additional file 1: Fig. S7a). At 0h, C/EBPα binds only to ~ 4% of significant interactions (either promoters or PIRs). After induction, C/EBPα binds to ~ 29% of significant interactions at 1h,  ~ 18% at 12h, and ~ 13% at 48h (Fig. 5a). We observed that PIRs have a significantly higher level of C/EBPα occupancy compared to random DNA controls (Fig. 5b). To investigate the association between differential C/EBPα binding and gene expression, we identified time-point unique and common C/EBPα binding sites at 1h, 12h, and 48h, compared to 0h (Additional file 1: Fig. S5b). We analyzed the expression fold change of genes with differential C/EBPα occupancy at their promoters. At 1h, 12h, and 48h post-C/EBPα induction, C/EBPα-occupied DEG promoters showed significantly increased expression (Fig. 5c). At 12h and 48h, increased C/EBPα occupancy at PIRs also correspond to increased expression levels of the DEGs they contact (Fig. 5d). For example, *Btg1* located on chr10, involved in cell growth and differentiation [60, 61], gradually gains contacts both upstream and downstream as trans-differentiation progresses (Fig. 5e). Several gained PIRs over a 490-kb region upstream of the *Btg1 *promoter show increased C/EBPα binding and gain of H3K27ac (Fig. 5e). The *Btg1* promoter itself is not occupied by C/EBPα, suggesting that the several C/EBPα-dependent enhancers in the region modulate *Btg1* expression through interaction with the *Btg1* promoter. Taken together, these results show numerous direct targets of C/EBPα binding to promoters and PIRs of differentially expressed genes, suggesting that changes in promoter interaction loops are a major driver of gene expression changes during Pre-B trans differentiation into macrophages.

## Discussion

Consistent with previous studies [34, 35], we observed efficient trans-differentiation of the C/EBPαER-expressing B cell line C10, providing us with a robust and reproducible system to investigate transcription factors driven chromosome reorganization during direct reprogramming from Pre-B cells to macrophages. Nuclear translocation of C/EBPα in Pre-B cells results in differential expression of thousands of genes. Transcription of B-cell-related genes is switched off and genes associated with macrophage functions and identity are switched on over the 48h period tested. We show that C/EBPα binding to thousands of sites including promoters and PIRs results in a rapid, significant change in the promoter interactome, with C/EBPα binding to 30% of significant promoter interactions genome-wide within 1h of induction. Genes with induced C/EBPα binding to their promoters are the first to respond showing significantly increased expression within 1h of induction. Genes whose promoters gain interactions with distal C/EBPα binding sites show delayed induction showing significantly increased expression at 12h. Our data also suggest widespread indirect effects of C/EBPα at other DEGs are associated with changes in enhancer-promoter interactions.

Altered gene expression during C/EBPα-induced trans-differentiation could not be explained by widespread changes in TAD or A/B compartment organization. We observed very high conservation of TAD boundary locations genome-wide with only a small percentage that shift location in association with the cell fate transition. Similarly, although some genes associated with macrophage functions, such as Gbp9 and Gbp4, switch from the B compartment to the A compartment during trans-differentiation, the vast majority of DEGs (94%) did not change compartment status over the 48h period. A recent study that used C/EBPα to trans-differentiate a human B cell line to macrophage also shows conservation of TAD boundaries, and observed only a small percentage of dynamic compartments associated with gene expression changes [62]. It is important to point out that these cells retain macrophage morphology, functions, and gene expression even after removal of estradiol indicating a stable change of cell fate [34, 63].

Our results suggest that the rewiring of promoter-anchored interactions plays a major role in controlling differential gene expression and cell fate transition compared to the relatively minor role played by changes in higher-order organization of TADs and A/B compartments. We show that PIRs are significantly enriched in histone modifications associated with gene regulatory activity. Genes whose promoters are contacted by PIRs with the active enhancer mark, H3K27ac, are highly expressed, while promoters contacted by the repressive H3K27me3 modification are not. Thus, transcription factor-mediated differential loop formation between promoters and their distal regulatory elements is the major driver of the cell-type-specific, promoter interactomes [50] and is the likely basis for tissue-specific gene expression profiles.

## Conclusions

Our integrated analyses of gene expression, histone modifications, transcription factor binding, and various levels of three-dimensional chromosome conformation during trans-differentiation have revealed that widespread changes in gene expression that characterize the transition from a Pre-B cell to a macrophage phenotype involve surprisingly few changes in TAD structure or A/B compartment status. Instead, we found reorganization of thousands of promoter-anchored loops, in conjunction with transcription factor binding and histone modification dynamics, occurring at thousands of differentially expressed genes suggesting that changes in chromatin interactions at the sub-TAD level are a major mechanism of differential regulation of gene expression that drives cell state transitions.

## Methods

### Cell culture, reprogramming induction, and flow cytometry

C10 cells were cultured in RPMI 1640 (ThermoFisher, 11875–093) medium supplemented with 10% heat-inactivated FBS (ThermoFisher, A3840202), 1% Penicillin–Streptomycin (ThermoFisher, 15140122), and 50 μM 2-Mercaptoethanol (ThermoFisher, 31350010). For C10 cells trans-differentiating into macrophages, 5.0 × 10^6^ C10 cells were plated in 10-cm cell culture dishes, followed by adding100 nM of β-estradiol (Sigma, E2758-250MG), 10 nM IL-3 (PEPRO TECH, 213–13), and CSF-1 (PEPRO TECH, 315–02). The cell culture medium was replenished every 24h. The Pre-B cell trans-differentiation efficiency was measured by flow cytometry analysis and qPCR gene expression analysis of Pre-B cell-specific genes and macrophage-specific genes. For flow cytometry analysis, C10 cells at various differentiation stages were stained using PE anti-mouse/human CD11b (BioLegend, 101208) antibody and APC anti-mouse CD19 antibody (BioLegend, 152410). The percentage of Pre-B cells and macrophage population was analyzed on FACSCanto II analyzer (BD Biosciences). Flow cytometry data was analyzed by FlowJo (TreeStar, Inc) software. PE Rat IgG2b (BioLegend, 400508) and APC Rat IgG2a (BioLegend, 400511) antibodies were used as isotype controls to exclude the background fluorescence.

### Western blot and immunofluorescence staining

For western blots, cells were lysed by CelLytic buffer (Millipore Sigma, C3228). The protein concentration was quantified by the Micro-BCA assay reagent (ThermoFisher Scientific, 23235). A 10 μg protein from each sample was loaded into 4–12% SDS–polyacrylamide (ThermoFisher Scientific, NP0321Box) for electrophoresis. The following antibodies were used for Western blot experiments: Anti-C/EBPα (Santa Cruz Biotechnology, AF2018), Anti-H3 (Cell Signaling Technology, 9715S), Anti-β-Tubulin (Cell Signaling Technology, 2128S). For immunofluorescence, C10 cells were first fixed in 4% PFA for 5 min, washed using cold PBS twice, followed by attaching cells to fibronectin and pre-coating the glass slides. Next, the cells were treated using blocking buffer (5% normal goat serum and 0.3% Triton X-100 in 1X PBS) for an hour at room temperature, followed by primary antibody treatment overnight at 4 °C and secondary antibody treatment at room temperature for an hour. The primary and secondary antibodies were diluted in antibody dilution buffer (1% BSA and 0.3% Triton X-100 in 1X PBS), and DAPI (Sigma Millipore, D9542) was used for nuclear staining. Anti-C/EBPα (Santa Cruz Biotechnology, AF2018) and Goat anti-Mouse IgG (H + L) Alexa Fluor 488 (ThermoFisher, A-1100A) antibodies were used for immunofluorescence staining. All the immunofluorescence images were taken by LSM 710 confocal microscope (Zeiss) and processed using Zen black (Zeiss).

### RNA extraction, qPCR assay of gene expression, RNA-seq libraries preparation, and sequencing

RNA was prepared from C10 cells at various trans-differentiation stages using RNeasy mini kit (Qiagen, Cat#74104) followed by on-column DNase I treatment. Total RNA (1 μg) was used for reverse transcription to generate cDNA by using iScript™ Reverse Transcription Supermix for RT-qPCR (BioRad, Cat#1708840), followed by the real-time PCR amplification using Power SYBR Green PCR Master Mix (Thermofisher SCIENTIFIC, Catalog number 4367659). The real-time PCR was performed with QuantStudio 7 Flex Real-Time PCR System (Thermofisher SCIENTIFIC) for 40 cycles, 95°C for 15 sec, and 60°C for 1 min. Gene expression was normalized to the housekeeping gene *Gapdh* using the 2^−ΔΔCT^ method.

For RNA-seq libraries preparation, 1μg of total RNA (RIN > 7) was used for rRNA depletion by using NEB rRNA Depletion Kit (E6310). Next, the NEBNext® Ultra™ II RNA Library Prep Kit for Illumina (E7770S) and NEBNext NEBNext® Multiplex Oligos for Illumina® (E7335) were used for library construction. RNA-seq library concentration was quantified by using the TapeStation and KAPA Library QANT Kit (Roche, 07960336001), followed by single-end (1 × 100bp) sequencing on the Novaseq S2 flow cell. The sequencing depth is described in Additional file 2: Table S1.

### Hi-C library and Promoter Capture Hi-C (PCHi-C) library preparation, sequencing

Hi-C and Promoter Capture Hi-C (PCHi-C) library preparation was described in our previous publication with minor modifications [20]. Ten million cells were used for each Hi-C library preparation. Briefly, we fixed cells using 2% formaldehyde for 10 min, followed by quenching in 0.125 M glycine for 5 min on ice. The fixed cells were washed twice in cold PBS and centrifuged at 760* g* for 5 min at 4°C. Next, the cells were lysed in 20 mL cold lysis buffer (10 mM Tris–HCl pH 8, 0.2% IGEPAL CA-630, 10 mM NaCl, and one tablet protease inhibitor cocktail) on ice for 30 min, followed by centrifuging at 760* g* for 5 min to remove the supernatant. The pellet was washed in 1.25X NEB buffer2 (NEB, B7002S) and resuspended in 358 μL 1.25X NEB buffer2, followed by adding 11 μL of 10% SDS and incubating at 37 °C with shaking at 950 rpm for 30 min. Lastly, the SDS was quenched by adding 75μL of 10% Triton X-100 and incubating at 37 °C for 15 min with shaking. To digest chromatin using HindIII restriction enzyme, we added 12 μL of 100 U/μL HindIII (NEB, R0104M) to each reaction, followed by shaking at 37 °C and 950 rpm overnight, followed by adding 5 μL of 100 U/μL HindIII (500 units in total) per reaction at 37 °C for 2 h on the following morning. To repair the digested overhangs, we added 6.1 μL 10X NEB buffer2, 25 μL molecular-grade water, 15.3 μL of 1 mM biotin-14-dATP (Jena Bioscience, NU-835-Bio14-L), 1.56 μL of 10 mM dCTP, 1.56 μL of 10 mM dGTP, 1.56 μL of 10 mM dTTP, and 10.2 μL of 5U/μL DNA polymerase I, Large (Klenow) Fragment (NEB, M0210L) and incubated the reaction at 37 °C for 1 h. In-nucleus ligation mix was prepared by adding 102μL 10X T4 DNA ligase buffer, 10.2 μL BSA (NEB, B9000S), 350.9 μL molecular-grade water, and 25.5 μL 1U/ μL T4 DNA ligase (ThermoFisher Scientific, 15224017), followed by incubation at 16 °C for 4 h in a thermomixer (shaking at 700 rpm for 5 s in every 2 min). Next, the nuclei were pelleted at 2500* g* for 5 min and resuspended in 300μL cross-link reversal buffer (10 mM Tris–HCl, 0.5 M NaCl, 1% SDS), followed by adding 5 μL 10 mg/ml RNase A (ThermoFisher Scientific, EN0531) at 37 °C for 30 min and 20 μL of 20 mg/ml Proteinase K (Gold Bio, P-480-SL2) at 55 °C for 1 h and 68 °C overnight. The genomic DNA was extracted using cold ethanol and sodium acetate (pH 5.2) and resuspended in 130μL of 10 mM Tris–HCl. DNA was transferred to a microTUBE AFA fiber Pre-Slit Snap-Cap (Covaris, 520045) and fragmented using a ME220 Covaris sonicator (Peak Incident Power 50W, Duty Factor 20%, Cycles per Burst 200, Treatment time 80 s). The sonicated DNA was transferred to a 1.5-ml tube, and the total volume was brought to 200 μL by adding 70 μL of molecular-grade water. DNA size was double size selected using AMPure XP beads (BECKMAN COULTER, A63881). First, 120 μL of beads were added to each reaction (ratio of AMPure beads to DNA: 0.6 to 1) and incubated for 5 min. The clear supernatant was transferred to a fresh tube through a magnetic separation stand. Next, 30 μl of fresh AMPure XP beads were added to the clear supernatant and incubated for 5 min at room temperature. Beads were separated on a magnetic separation stand and washed twice using 800 μL of 70% ethanol and dried at 37 °C, followed by eluting DNA in 300 μL 1X Tris buffer. DNA was used for library preparation with a normal size distribution between 300 and 500 bp and was visualized by TapeStation.

Procedures for Biotin/Streptavidin pull-down of the Hi-C ligation products, end repair, removal of biotin at the non-ligated DNA ends, adaptors ligation, Hi-C libraries amplification, and Hi-C size selection were described in our recent publication [64]. The Hi-C library size distribution was evaluated by using TapeStation, and the Hi-C library concentration was estimated by using the Qubit 4 Fluorometer and KAPA Library QANT Kit (Roche, E7770S). Procedures and baited capture system design for Promoter Capture Hi-C libraries preparation were described in our previous publication [19, 20]. 39,021 biotinylated RNA bait target 22,225 annotated gene promoters that include protein-coding, non-coding(lincRNA), antisense, snRNA, miRNA, or snoRNA. Two unique 120 bp capture probes were designed close to the ends of each restriction fragment (one to each end) containing a transcription start site. In rare instances, a unique sequence could not be found for one end, or even more rare, for both ends. For each reaction, 500 ng of dried Hi-C DNA was used for library preparation. SureSelectXT Custom 3–5.9 Mb (Agilent, 5190–4831) and SSEL TE Reagent Kit, ILM PE FULL Adaptor (Agilent, 931108) were used for enriching the Hi-C fragment that is associated with promoters. The procedures for Promoter Capture Hi-C libraries amplification were described in detail in our recent publication [64]. The Promoter Capture Hi-C library size distribution was evaluated by using TapeStation, and the library concentration was quantified by using the Qubit 4 Fluorometer and KAPA Library QANT Kit (Roche, E7770S). Hi-C and Promoter Capture Hi-C libraries were paired-end sequenced (2 × 50 bp) on Novaseq S2 flow cells. The sequencing depth is described in Additional file 1: Table S1.

### RNA-seq data processing

RNA-seq libraries were prepared for each time point. We included three biological and four technical replicates for each time point. Technical replicates were merged, and Trim Galore (0.6.2) pipeline was used to remove adapters and short reads from the data (https://github.com/FelixKrueger/TrimGalore). FastQC program was used to check the quality of the data (http://www.bioinformatics.babraham.ac.uk/projects/fastqc/). Trimmed reads were mapped to mouse reference genome (mm10) using Hisat2 (2.1.0) with default parameters [65]. Samtools (1.10) were used to convert the sam files to bam files [66]. GTF file (GRCm38.96) was used to annotate the mapped reads using featureCounts tool [67]. Exon was chosen as the feature gene type with a default minimum overlap of 1 bp. The genes with the sum of read number in all samples less than 2 were removed from further analysis. Differentially expressed genes (DEGs) were identified using R package DEseq2 by keeping filtering thresholds of Padj < 0.05 and fold change > 2 [68]. Sample distances were assessed and visualized by the principal components analysis (PCA) function in R. TPM (transcripts per million) values of each gene were obtained using RSEM [69]. Fuzzy c-means clustering was performed on DEGs using R [70]. GO analysis was performed on WebGestalt toolkit with “over-representation analysis” as the method of interest and “biological process” as the functional database [71]. SNPsplit was used to analyze the expression of the rat Cebpa transgene and endogenous mouse Cebpa gene [72].

### Hi-C data analysis

Hi-C data were processed using HiCUP (v0.7.2) pipeline [73] with mm10 as the reference genome. Hi-C interaction matrices’ reproducibility of the biological replicates was assessed by Hi-CRep (v1.8.0) using the stratum-adjusted correlation coefficient method [74]. Hi-CUP bam files were converted to Hi-C files using Hi-Cup2homer and tagDir2Hi-CFile.pl script in HiCUP and Homer package(http://homer.ucsd.edu/homer/). JuiceBox was used to visualize Hi-C matrix heatmap and TAD structure [75]. Homer package was used to call the A/B compartment at 50-kb resolution [76]. The correlation coefficient of PC1 value was calculated to assess the reproducibility of biological replicates. A/B compartment switches were estimated based on PC1 values. To calculate the correlation between DEGs and A/B compartment switch, A/B switch was classified into four categories: A to B, B to A, A stable, and B stable. DEGs were assigned to each category based on overlap between the start coordinates of DEGs and compartment bin. The A/B compartment was visualized using Integrative Genomics Viewer (IGV) [77]. Hi-C files were converted to h5 format at 50-kb resolution, and KR correction was performed using Hi-CExplorer (v3.3) tool [78]. TADs and TAD boundaries were defined by Hi-CExplorer at 50-kb resolution with parameters (*Hi-CFindTADs –correctForMultipleTesting fdr –thresholdComparisons 0.01 –delta 0.01*) [78].

### PCHi-C interaction analysis

PCHi-C mouse raw reads were processed by the HiCUP pipeline and were mapped to mm10 genome [73]. Hi-CRep was applied to assess the PCHi-C interaction matrices’ reproducibility of the biological replicates [74]. CHICAGO (v1.12.0) pipeline was used to call the significant interactions with CHiCAGO score cut-off 5 [79]. PCHi-C contacts were visualized using WashU Browser (https://epigenomegateway.wustl.edu/) [80]. To estimate the correlation between gene expression and the PCHi-C interactions, the expression level was classified into 4 categories: TPM = 0, TPM = 0–1, TPM = 1–5, and TPM > 5. The enrichment of markers (H3K27ac, H3K4me1, p300, and H3K27me3) on promoter interacting regions (PIRs) was tested by shuffling the distance-matched fragments around the genome as control (*peakEnrichemnt4Features* function in CHICAGO) [79].

BEDTools (v2.26.0) “intersect” function was used to find PIR regions that overlap with H3K27ac peaks or H3K27me3 [81]. To investigate the association between promoter expression level and its PIRs, promoters were categorized into four groups based on whether at least one PIR overlapped with H3K27ac or H3K27me3 peaks and compared the TPM values of the respective promoters. The contacts showing significant changes at different time points were identified by running Chicdiff (v0.5) package with default setting [57]. When assessing the association between alteration of interactions and regulation of gene expression, promoters were divided into two groups based on whether their contact frequency significantly increased or significantly decreased. Pheatmap function in R was used to cluster differential interactions with a score greater than or equal to 5 in at least one time point based on the Euclidean distance for CHICAGO scores across all time points [70].

### ChIP-seq data analysis

ChIP-seq data C/EBPα along with histone modification ChIP data were downloaded from GEO [37]. Raw reads were mapped end-to-end to the mm10 reference genome using Bowtie2 [82]. SAM format files were converted to BAM format and were sorted by Samtools [66]. The *findPeaks* function of HOMER package was used to call histone modification peaks by setting peak size 1000 bp and the remaining parameters were set to the default values [76]. Peak calling for C/EBPα data was performed using MACS2 (v2.1.1) (minimum FDR cutoff 0.01) [83]. To visualize the peak, peak files were loaded to IGV [77]. The enrichment analysis of C/EBPα binding peaks on PIRs was performed by running CHICAGO *peakEnrichmen4Features* [79]. Further, to quantitatively compare differential binding sites of C/EBPα at different time points after induction, MAnorm (v1.3.0) package was used with default settings [84].* p*-value < 0.05 and fold change > 1.5 were used as threshold to identify induced C/EBPα binding sites. TSS of all genes were downloaded from Ensembl biomart (v100) [85]. Bedtools intersect was used to find at least 1-bp overlap between differential C/EBPα binding sites and TSSs (a total of 2–1 kb upstream and 1 kb downstream of TSS) or PIRs [81].

### Statistical analysis

Unpaired *t*-test, Mann–Whitney *U* statistical test, and chi-squared test were performed using GraphPad Prism 8 and R. *p*-value < 0.05 was considered as significant unless specified and indicated as * *p* < 0.05, ** *p* < 0.01 and *** *p* < 0.001.

### Supplementary Information

**Additional file 1:** **Fig. S1.** Transcriptional profile changes during Pre-B transdifferentiation. **a** Principal component analysis (PCA) RNAseq of each replicate from different time points. **b** SNPsplit identifies the percentage of total RNA reads that align to Cebpa gene region that contain Mus musculus specific SNP (mouse specific), Rat specific SNP (Rat specific), regions that do not contain any SNP (Unassignable), conflicting SNP information (Conflicting). **c **Volcano plots show upregulated and downregulated differentially expressed genes (DEGs).** d **GO ontology 5 Fuzzy c-means clusters of DEGs **e **qPCR analysis of Pre-B cell and macrophage marker gene expression during Pre-B cell trans-differentiation.** f **Gene expression level (TPM) of Pre-B cell-specific genes (*Blnk, Cd19, Cd79a, Cd79b, Vpreb1, Vpreb2, Vpreb3*), and macrophage-specific genes (*Ccl6, Ctsc, Mmp8, Msr1*) during Pre-B trans-differentiation. **Fig. S2.** Hi-C data analysis. **a**HiCUP pipeline analysis HiC and PCHi-C data, including the following three categories: *Cis* < 10Kbp, *Cis* > 10Kbp, and *trans*-interactions across four-time points. **b** Heatmaps show Hi-C matrix at 100kb and 25 kb resolution. **Fig. S3.** Altered TAD and A/B compartment during Pre-B transdifferentiation. **a** Number of TADs and size distribution at each time point. **b **GO enrichment analysis of genes changed TAD boundaries from 0h to 48h. **c** Pearson correlation coefficient of PC1 values (50kb resolution) of the entire genome. **d **Gene expression level (TPM) in the A and B compartments at four different time points. A two-sided unpaired t-test was performed for the significance test. **e**Percentage of compartment change for DEGs (differentially expressed genes)**. ** **f **Log2 fold change of gene expression in dynamic compartments. stable (*n*= 19157), A to B (*n*= 450), and B to A (*n*= 474). **g **Gene ontology analysis of 2105 genes at 48h that switch from A to B and 1552 genes at 48h that switch from B to A. A two-sided unpaired t-test was performed for the significance test. **Fig. S4. **PCHi-C data analysis.** a** HiCUP pipeline analysis of PCHi-C data: *Cis* <10Kbp, *Cis* >10Kbp, and *trans*-interactions across four-time points. **b **Proportion of promoter-promoter (P-P) and promoter-other interactions (P-O) interactions as deduced from PCHi-C. **c **Expression values (log2(TPM+1)) of genes/promoters classified into 4 groups (at least one PIR overlaps with histone markers). H3K27ac: interaction between gene/promoter and PIRs only with H3K27ac marks. 0h (*n*=4829); 1h (*n*=5710);12h (*n*=5257); 48h (5735). H3K27Ac+H3K27me3: interaction between gene/promoter and PIRs with both H3K27ac and H3K27me3 marks. 0h (*n*=8075); 1h (*n*=7044); 12h (*n*=7926); 48h (*n*= 7727). H3K27me3: interaction between gene/promoter and PIRs only with H3K27me3. 0h (*n*=2757);1h (*n*=2286); 12h (*n*= 2545); 48h (*n*=2165). No marks: interaction between gene/promoter and PIRs without any histone modification. 0h (*n*=3667), 1h (*n*=4805); 12h (*n*=4403); 48h (*n*= 4291). The box plot represents 25 and 75 percentiles with the median. An unpaired two-sided t test is performed for the significance test. **d **Boxplot shows gene expression values (log2(TPM +1)) of promoters that do not contact any PIRs with H3K27ac categorized into four groups according to the number of PIRs marked by H3K27me3 at four different time points.  0: Promoters without any PIRs marked by H3K27me3. 0h (*n*=2551), 1h (*n*=2991); 12h (*n*=2711); 48h (*n*=2682).  1: Promoters with only one PIR marked by H3K27me3 0h (*n*=1247), 1h (*n*=1100); 12h (*n*=1188); 48h (*n*= 955). 1-5: Promoters with one to five PIRs marked by H3K27me3. 0h (*n*=749), 1h (*n*=672); 12h (*n*=697); 48h (*n*=567).  >5: Promoters with more than five PIRs marked by H3K27me3. 0h (*n*=80), 1h (*n*=63); 12h (*n*=103); 48h (*n*=95).  An unpaired two-sided t-test is performed for the significance test. The box plot represents 25 and 75 percentiles with the median. **Fig. S5.** Integrated analysis of promoter interactions and epigenetic features. **a ***Maf1* and *Ssbp1 *region that shows promoter interaction changes accompanied by H3K27me3(green), H3K27ac (yellow), and H3K4me1 at interacting regions, along with gene expression TPM of *Maf1* and *Ssbp1* during Pre-B transdifferentiation. Multiple *Maf1* promoters in two HindIII digested fragments **b** Log2 fold change of gene expression based on the number of H3K4me1 peaks on the increased PIRs (48h vs 0h). The number of histone mark peaks is classified into three groups.  <=1: Genes that gain chromatin interactions with less than one histone mark peaks (*n*=360). 1-5:  Genes that gain chromatin interactions with one to five histone mark peaks (*n*=313). >5: Genes that gain chromatin interactions with greater than five histone mark peaks (*n*=69). The unpaired two-sided t-test was performed to test significant differences between groups. The box plot represents 25 and 75 percentiles with the median. **Fig. S6.** Decreased promoter interactions at 48h. **a **GO term for genes that lost promoter interactions at 48h. **b **Examples of significantly decreased B cell-specific gene promoter interactions (Pax5,and *Clcf1*) across time points. **Fig. S7.** C/EBPα ChIPseq data analysis. **a **The number of identified C/EBPα ChIP peaks at 1h, 1h, 12 h and 48h. **b **Comparison of 1h, 12,h or 48h C/EBPα ChIP peaks signal to 0h. M value represents log2 fold change of read density between two samples, and A value represents the average signal intensity. The red dots represent induced C/EBPα binding sites at 1h, 12h, and 48h time points. The blue dots represent C/EBPα binding sites at 0h. The grey dots represent unchanged C/EBPα binding sites at four time points. **Fig. S8. **Uncropped blots.

**Additional file 2:** **Table S1.**

**Additional file 3:** **Table S2.**

**Additional file 4:** **Table S3.**

## Data Availability

Histone modifications ChIP-seq data is available at GEO with accession number GSE53173 [86]. C/EBPα ChIP-seq data is available at GEO with accession number GSE53362 [87]. RNA-seq, Hi-C, and Promoter Capture Hi-C data of Pre-B cell differentiation were deposited in Gene Expression Omnibus: GSE221737 [88].

## Abbreviations

PCHi-C: Promoter capture Hi-C
TADs: Topological associated domains
ESC: Embryonic stem cell
HiChIP: Highly integrative chromatin immunoprecipitation
H3: Histone 3
PCA: Principal component analysis
DEGs: Differentially expressed genes
GO: Gene ontology
