# Supplementary material for: Cellular reprogramming is driven by widespread rewiring of promoter-enhancer interactions
Source: BMC Biol. 2023 Nov 20;21:264. doi: 10.1186/s12915-023-01766-0 (PMC10658794; doi:10.1186/s12915-023-01766-0)
Supplement: Supplementary file 1 — Additional file 1: Fig. S1. Transcriptional profile changes during Pre-B transdifferentiation. a Principal component analysis (PCA) RNAseq of each replicate from different time points. b SNPsplit identifies the percentage of total RNA reads that align to Cebpa gene region that contain Mus musculus specific SNP (mouse specific), Rat specific SNP (Rat specific), regions that do not contain any SNP (Unassignable), conflicting SNP information (Conflicting). c Volcano plots show upregulated and downregulated differentially expressed genes (DEGs). d GO ontology 5 Fuzzy c-means clusters of DEGs e qPCR analysis of Pre-B cell and macrophage marker gene expression during Pre-B cell trans-differentiation. f Gene expression level (TPM) of Pre-B cell-specific genes (Blnk, Cd19, Cd79a, Cd79b, Vpreb1, Vpreb2, Vpreb3), and macrophage-specific genes (Ccl6, Ctsc, Mmp8, Msr1) during Pre-B trans-differentiation. Fig. S2. Hi-C data analysis. aHiCUP pipeline analysis HiC and PCHi-C data, including the following three categories: Cis < 10Kbp, Cis > 10Kbp, and trans-interactions across four-time points. b Heatmaps show Hi-C matrix at 100kb and 25 kb resolution. Fig. S3. Altered TAD and A/B compartment during Pre-B transdifferentiation. a Number of TADs and size distribution at each time point. b GO enrichment analysis of genes changed TAD boundaries from 0h to 48h. c Pearson correlation coefficient of PC1 values (50kb resolution) of the entire genome. d Gene expression level (TPM) in the A and B compartments at four different time points. A two-sided unpaired t-test was performed for the significance test. ePercentage of compartment change for DEGs (differentially expressed genes). f Log2 fold change of gene expression in dynamic compartments. stable (n= 19157), A to B (n= 450), and B to A (n= 474). g Gene ontology analysis of 2105 genes at 48h that switch from A to B and 1552 genes at 48h that switch from B to A. A two-sided unpaired t-test was performed for the significance test. Fig. S4 [file 12915_2023_1766_MOESM1_ESM.pdf]

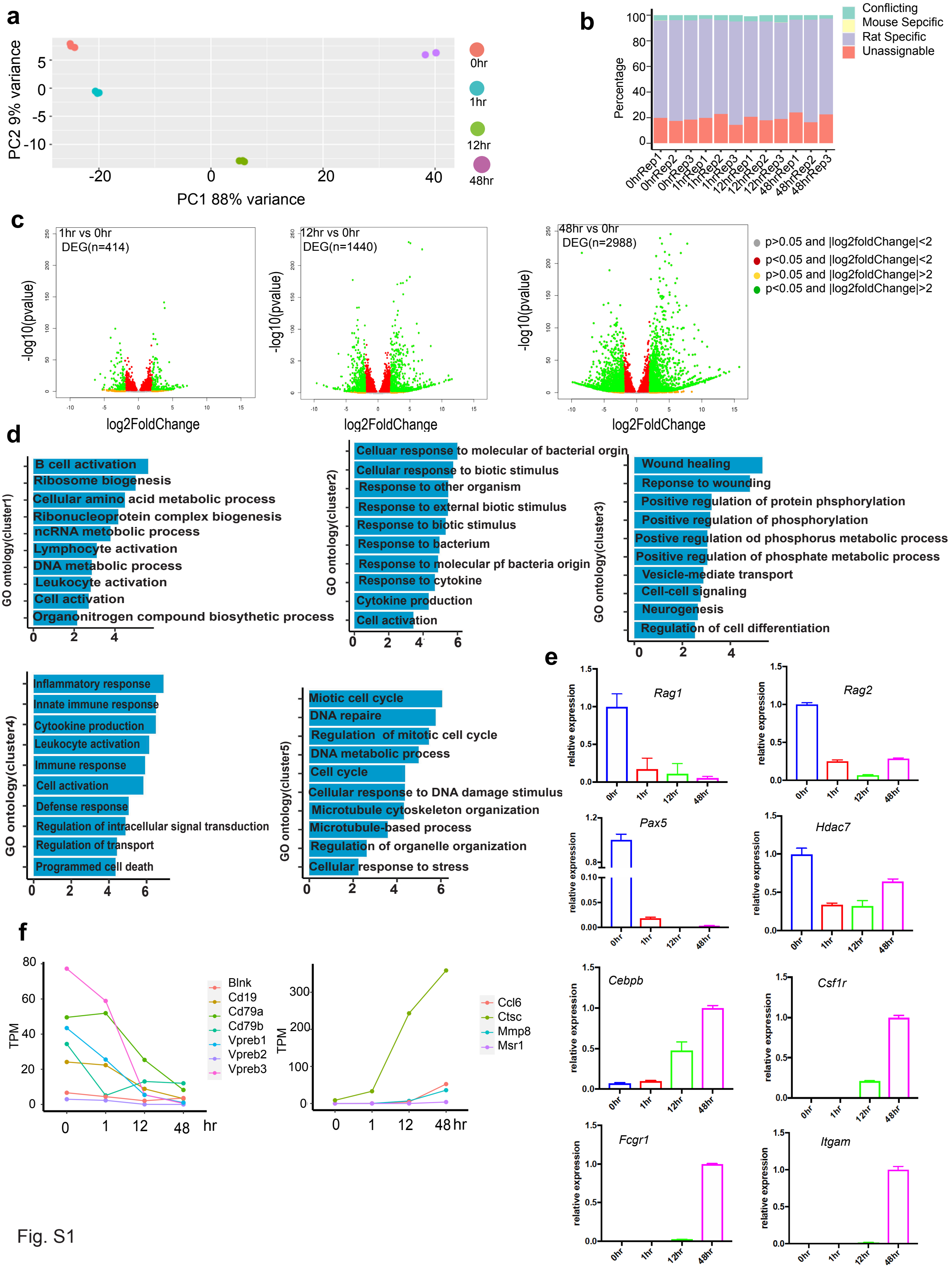

a
PC1 88% variance
PC2 9% variance
0hr
1hr
12hr
48hr
b
Conflicting
Mouse Sepcific
Rat Specific
Unassignable
Percentage
0hrRep1
0hrRep2
0hrRep3
1hrRep1
1hrRep2
1hrRep3
12hrRep1
12hrRep2
12hrRep3
48hrRep1
48hrRep2
48hrRep3
c
1hr vs 0hr
DEG(n=414)
12hr vs 0hr
DEG(n=1440)
48hr vs 0hr
DEG(n=2988)
-log10(pvalue)
log2FoldChange
p>0.05 and |log2foldChange|<2
p<0.05 and |log2foldChange|<2
p>0.05 and |log2foldChange|>2
p<0.05 and |log2foldChange|>2
d
GO ontology(cluster1)
B cell activation
Ribosome biogenesis
Cellular amino acid metabolic process
Ribonucleoprotein complex biogenesis
ncRNA metobolic process
Lymphocyte activation
DNA metabolic process
Leukocyte activation
Cell activation
Organonitrogen compound biosythetic process
GO ontology(cluster2)
Cellular response to molecular of bacterial orgin
Cellular response to biotic stimulus
Response to other organism
Response to external biotic stimulus
Response to biotic stimulus
Response to bacterium
Response to molecular pf bacteria origin
Response to cytokine
Cytokine production
Cell activation
GO ontology(cluster3)
Wound healing
Reponse to wounding
Positive regulation of protein phsphorylation
Positive regulation of phosphorylation
Postive regulation od phosphorus metabolic process
Positive regulation of phosphate metabolic process
Vesicle-mediate transport
Cell-cell signaling
Neurogenesis
Regulation of cell differentiation
GO ontology(cluster4)
Inflammatory response
Innate immune response
Cytookine production
Leukocyte activation
Immune response
Cell activation
Defense response
Regulation of intracellular signal transduction
Regulation of transport
Programmed cell death
GO ontology(cluster5)
Miotic cell cycle
DNA repaire
Regulation of mitotic cell cycle
DNA metabolic process
Cell cycle
Cellular response to DNA damage stimulus
Microtubule cytoskeleton organization
Microtubule-based process
Regulation of organelle organization
Cellular response to stress
e
Rag1
Rag2
Pax5
Hdac7
Cebpb
Csf1r
Fcgr1
Itgam
relative expression
0hr
1hr
12hr
48hr
f
TPM
Blnk
Cd19
Cd79a
Cd79b
Vpreb1
Vpreb2
Vpreb3
Ccl6
Ctsc
Mmp8
Msr1
hr

Fig. S1

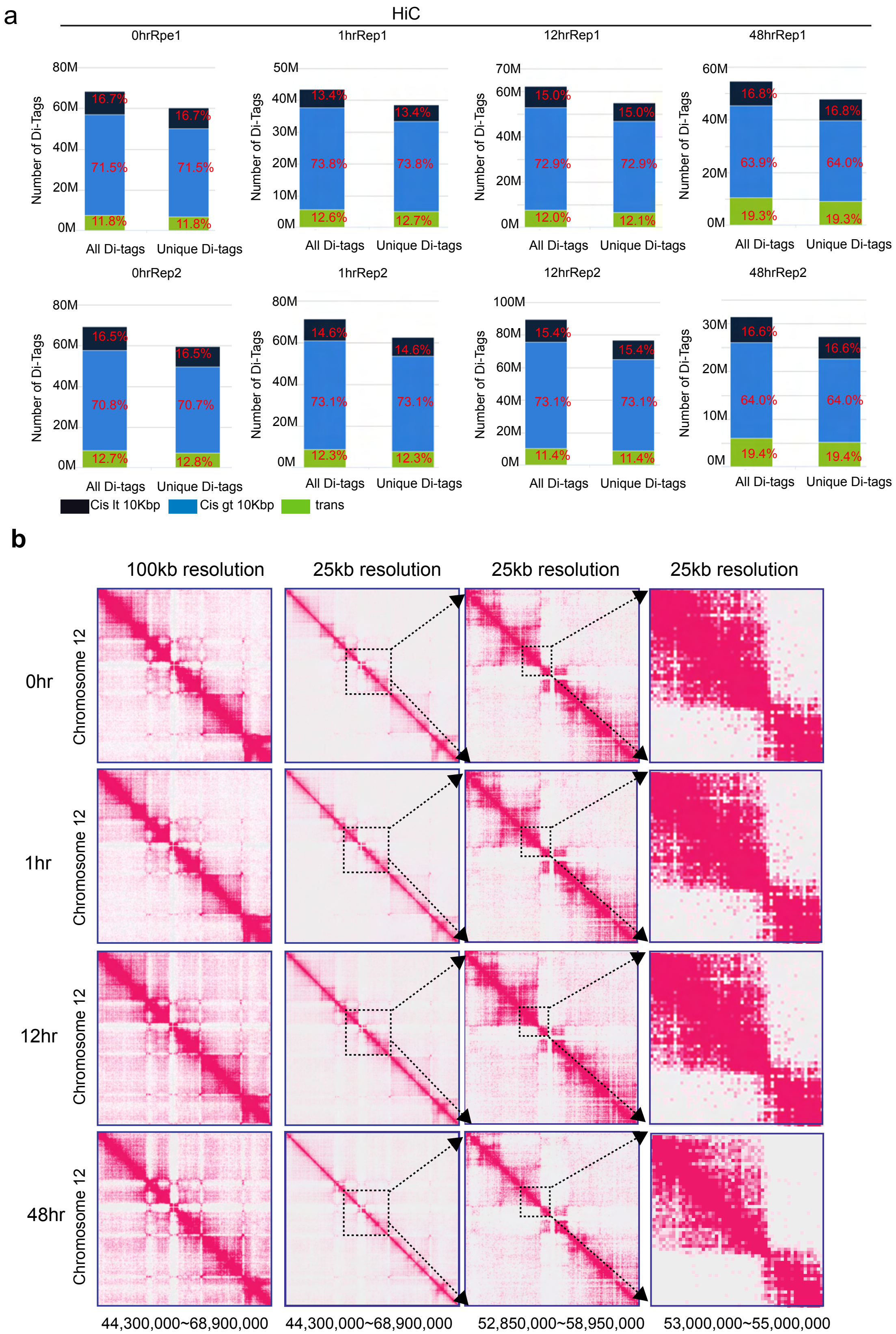
a
HiC
0hrRpe1
1hrRep1
12hrRep1
48hrRep1
Number of Di-Tags
All Di-tags
Unique Di-tags
0hrRep2
1hrRep2
12hrRep2
48hrRep2
Cis lt 10Kbp
Cis gt 10Kbp
trans
b
100kb resolution
25kb resolution
25kb resolution
25kb resolution
0hr
1hr
12hr
48hr
Chromosome 12
44,300,000~68,900,000
44,300,000~68,900,000
52,850,000~58,950,000
53,000,000~55,000,000

Fig. S2

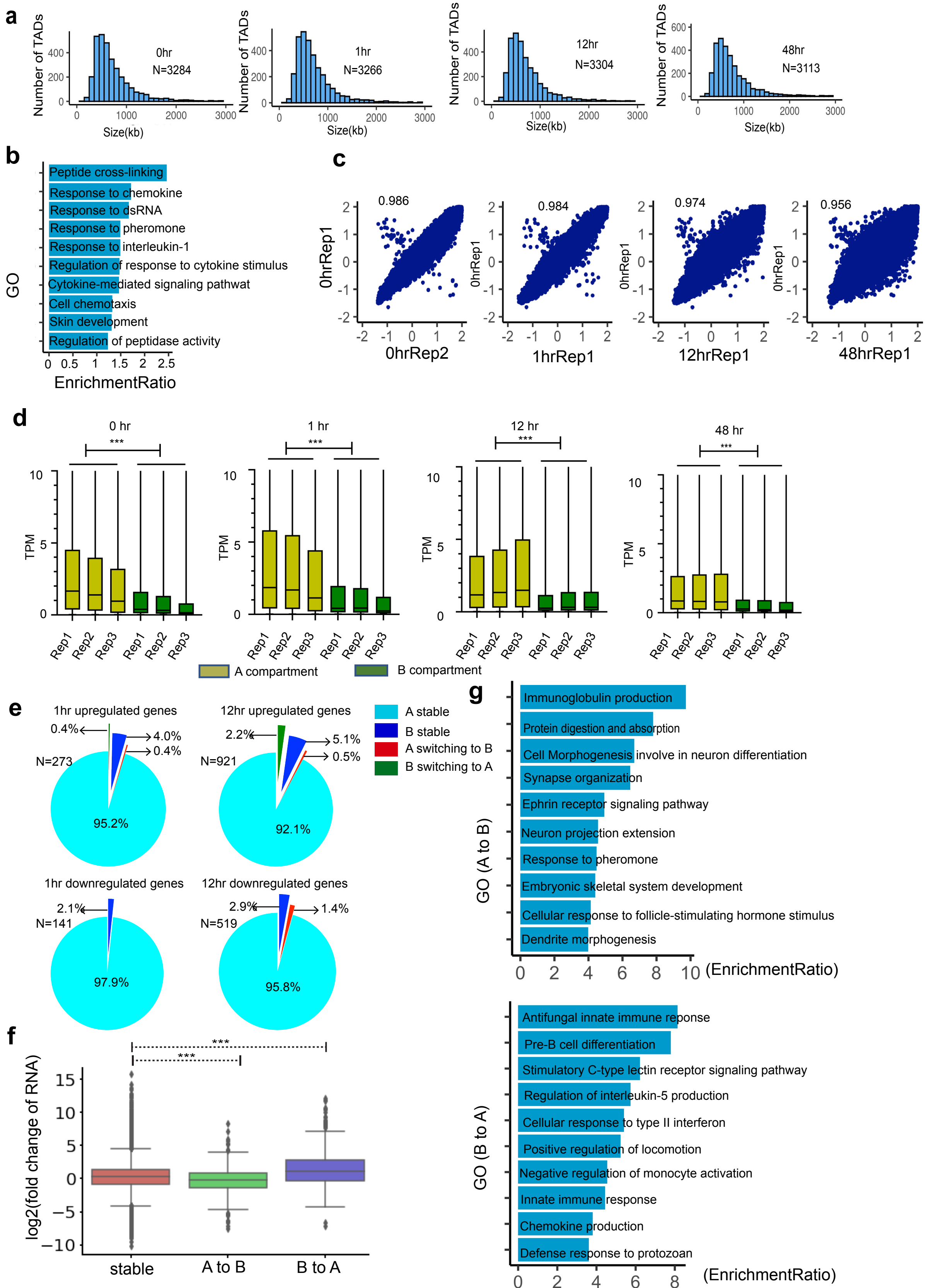

a
0hr N=3284
1hr N=3266
12hr N=3304
48hr N=3113
Number of TADs
Size(kb)
b
Peptide cross-linking
Response to chemokine
Response to dsRNA
Response to pheromone
Response to interleukin-1
Regulation of response to cytokine stimulus
Cytokine-mediated signaling pathwat
Cell chemotaxis
Skin development
Regulation of peptidase activity
GO
EnrichmentRatio
c
0.986
0.984
0.974
0.956
0hrRep1
0hrRep2
1hrRep1
12hrRep1
48hrRep1
d
0 hr
1 hr
12 hr
48 hr
TPM
Rep1
Rep2
Rep3
A compartment
B compartment
e
1hr upregulated genes
N=273
0.4%
4.0%
0.4%
95.2%
12hr upregulated genes
N=921
2.2%
5.1%
0.5%
92.1%
1hr downregulated genes
N=141
2.1%
97.9%
12hr downregulated genes
N=519
2.9%
1.4%
95.8%
A stable
B stable
A switching to B
B switching to A
f
log2(fold change of RNA)
stable
A to B
B to A
g
Immunoglobulin production
Protein digestion and absorption
Cell Morphogenesis involve in neuron differentiation
Synapse organization
Ephrin receptor signaling pathway
Neuron projection extension
Response to pheromone
Embryonic skeletal system development
Cellular response to follicle-stimulating hormone stimulus
Dendrite morphogenesis
GO (A to B)
(EnrichmentRatio)
Antifungal innate immune reponse
Pre-B cell differentiation
Stimulatory C-type lectin receptor signaling pathway
Regulation of interleukin-5 production
Cellular response to type II interferon
Positive regulation of locomotion
Negative regulation of monocyte activation
Innate immune response
Chemokine production
Defense response to protozoan
GO (B to A)
(EnrichmentRatio)

Fig. S3

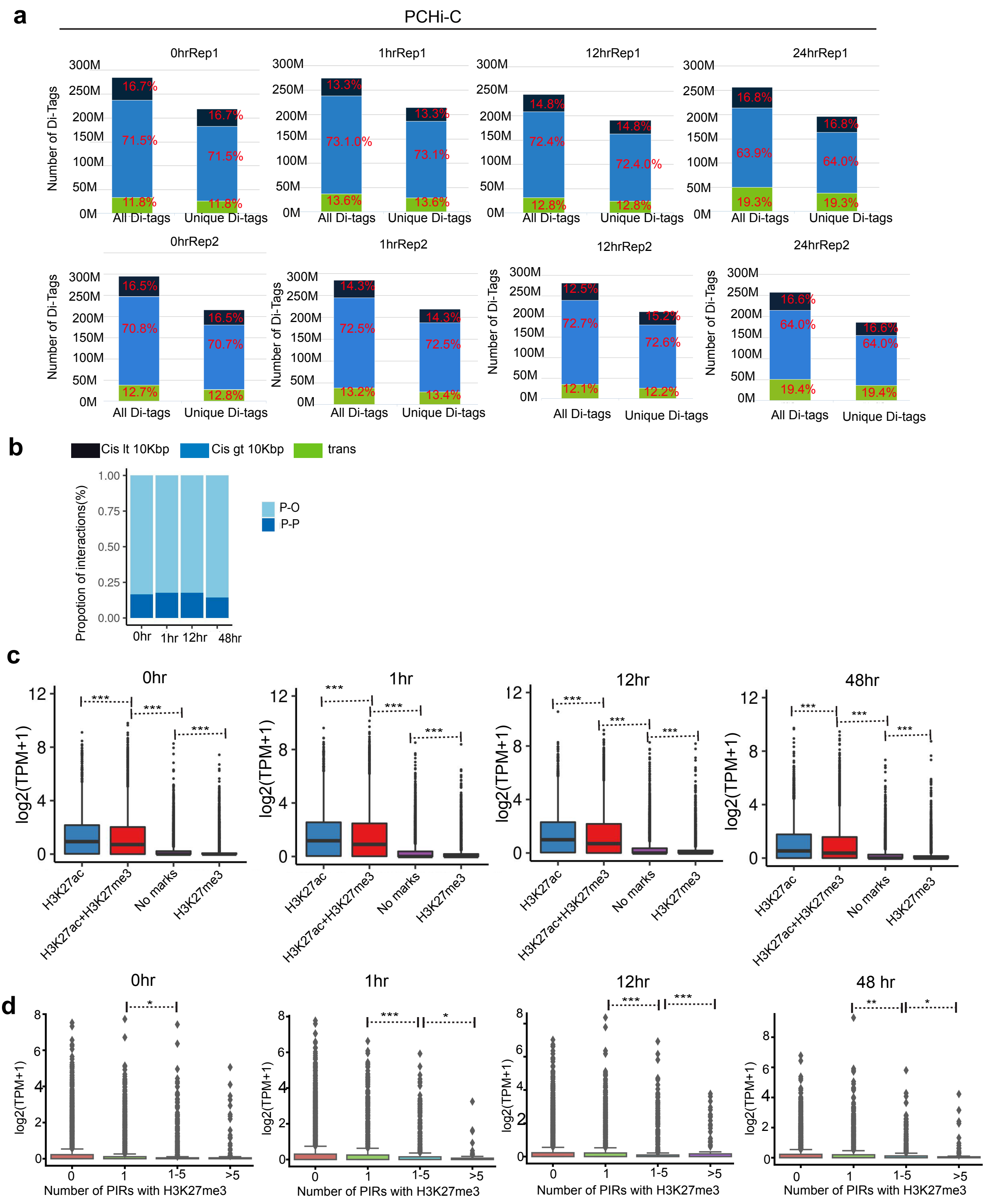
a
PCHi-C
0hrRep1
1hrRep1
12hrRep1
24hrRep1
0hrRep2
1hrRep2
12hrRep2
24hrRep2
Number of Di-Tags
300M
250M
200M
150M
100M
50M
0M
All Di-tags
Unique Di-tags
b
Cis lt 10Kbp
Cis gt 10Kbp
trans
Propotion of interactions(%)
1.00
0.75
0.50
0.25
0.00
0hr
1hr
12hr
48hr
P-O
P-P
c
0hr
1hr
12hr
48hr
log2(TPM+1)
H3K27ac
H3K27ac+H3K27me3
No marks
H3K27me3
d
0hr
1hr
12hr
48 hr
log2(TPM+1)
0
1
1-5
>5
Number of PIRs with H3K27me3

Fig S4

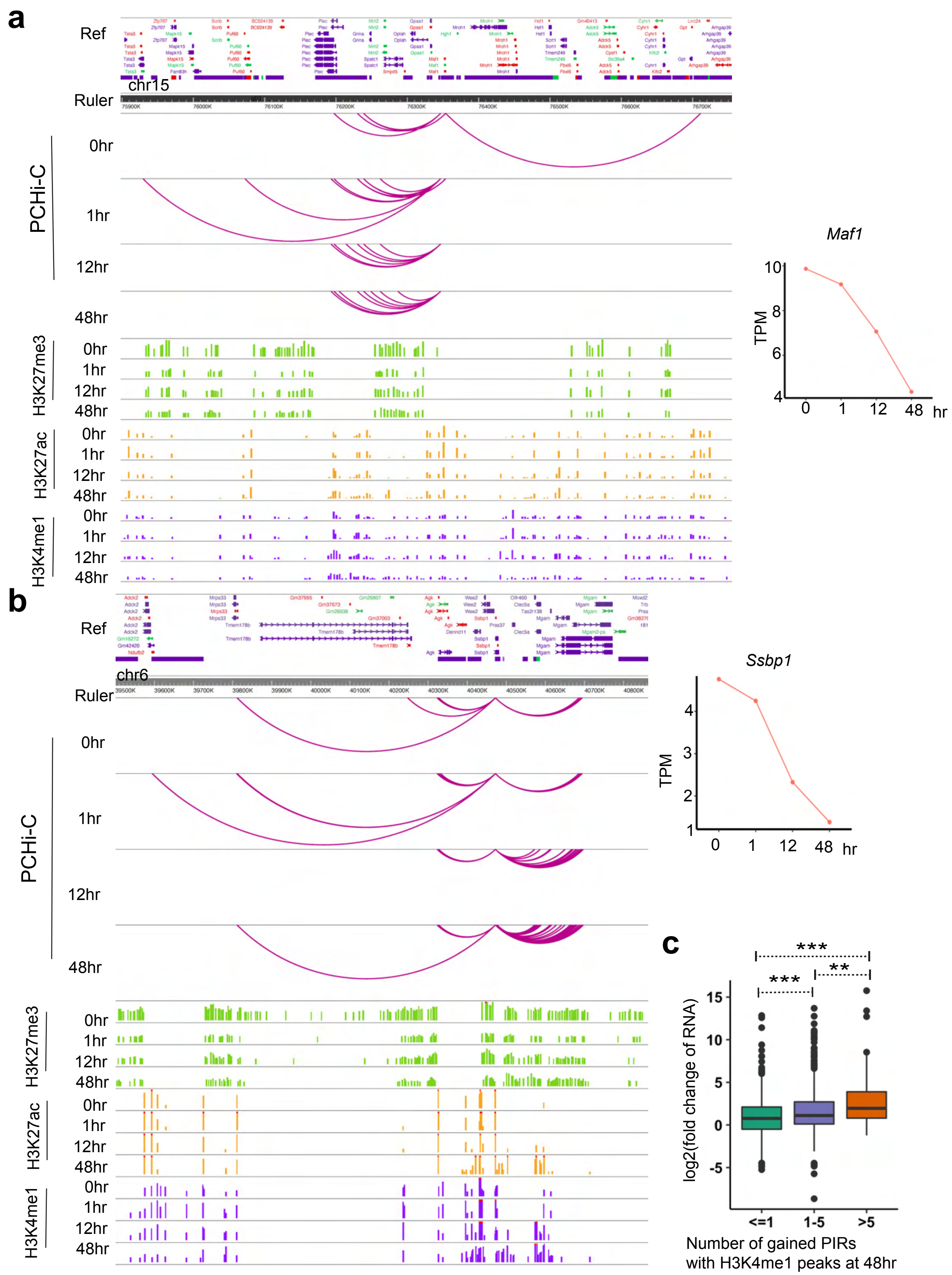
a
Ref
chr15
Ruler
PCHi-C
0hr
1hr
12hr
48hr
H3K27me3
H3K27ac
H3K4me1
Maf1
TPM
10
8
6
4
0
1
12
48
hr
b
Ref
chr6
Ruler
PCHi-C
Ssbp1
TPM
4
3
2
1
0
1
12
48
hr
c
***
***
**
log2(fold change of RNA)
15
10
5
0
-5
<=1
1-5
>5
Number of gained PIRs
with H3K4me1 peaks at 48hr

Fig. S5

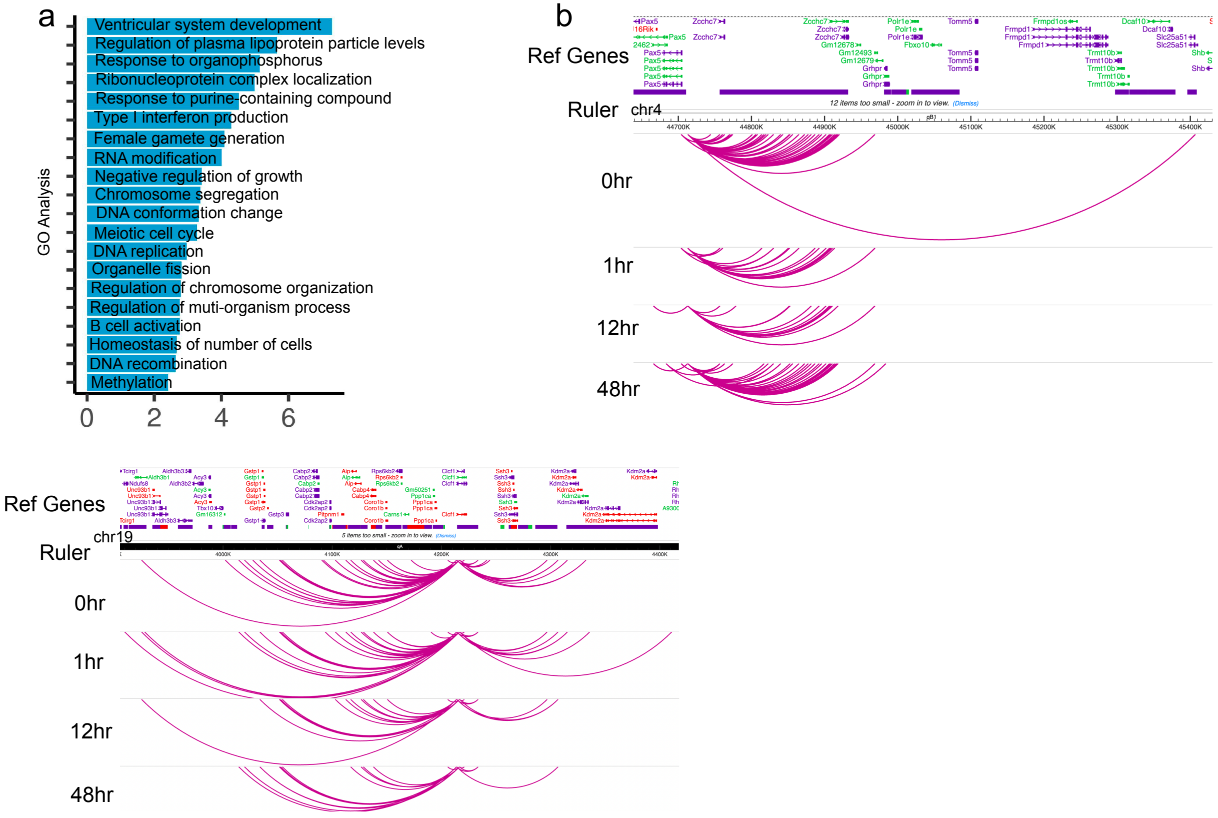

a
GO Analysis
Ventricular system development
Regulation of plasma lipoprotein particle levels
Response to organophosphorus
Ribonucleoprotein complex localization
Response to purine-containing compound
Type I interferon production
Female gamete generation
RNA modification
Negative regulation of growth
Chromosome segregation
DNA conformation change
Meiotic cell cycle
DNA replication
Organelle fission
Regulation of chromosome organization
Regulation of muti-organism process
B cell activation
Homeostasis of number of cells
DNA recombination
Methylation
0
2
4
6
b
Ref Genes
Ruler
chr4
0hr
1hr
12hr
48hr
Ref Genes
Ruler
chr19
0hr
1hr
12hr
48hr

Fig. S6

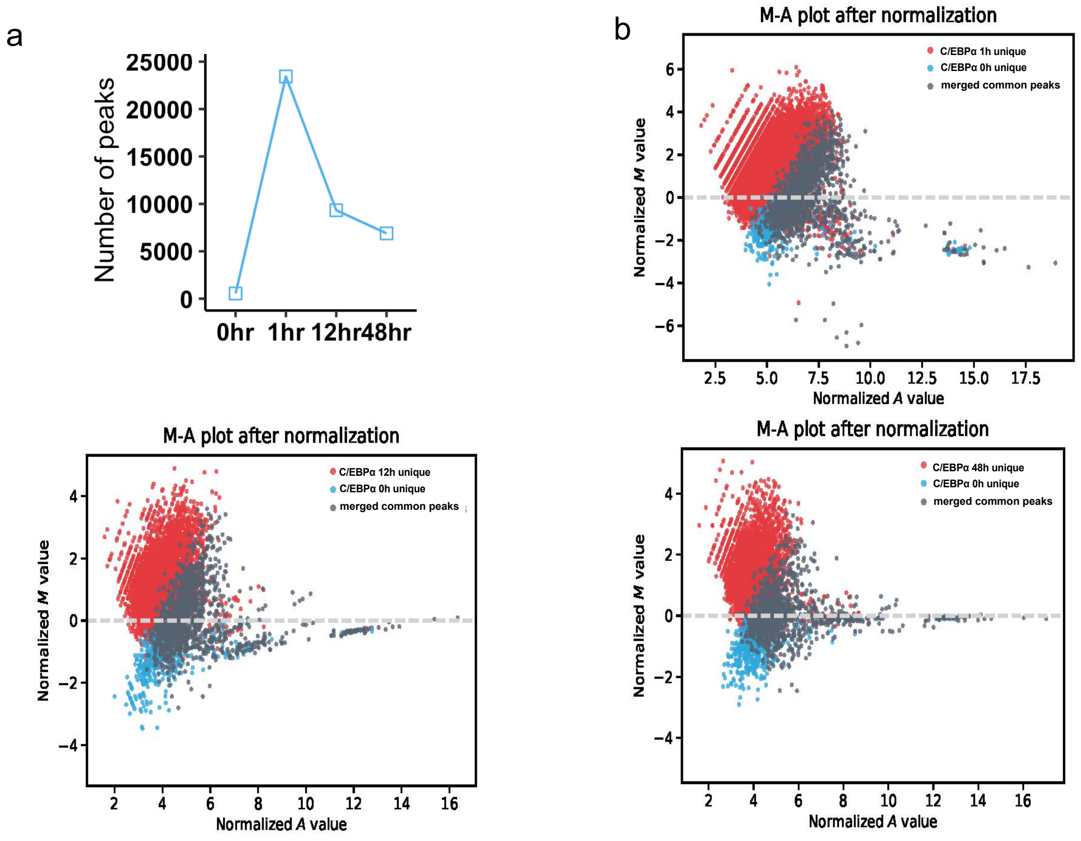
a
Number of peaks
25000
20000
15000
10000
5000
0
0hr
1hr
12hr
48hr
b
M-A plot after normalization
C/EBPα 1h unique
C/EBPα 0h unique
merged common peaks
Normalized M value
Normalized A value
M-A plot after normalization
C/EBPα 12h unique
C/EBPα 0h unique
merged common peaks
Normalized M value
Normalized A value
M-A plot after normalization
C/EBPα 48h unique
C/EBPα 0h unique
merged common peaks
Normalized M value
Normalized A value

Fig. S7

Western blot results for Fig. 1e

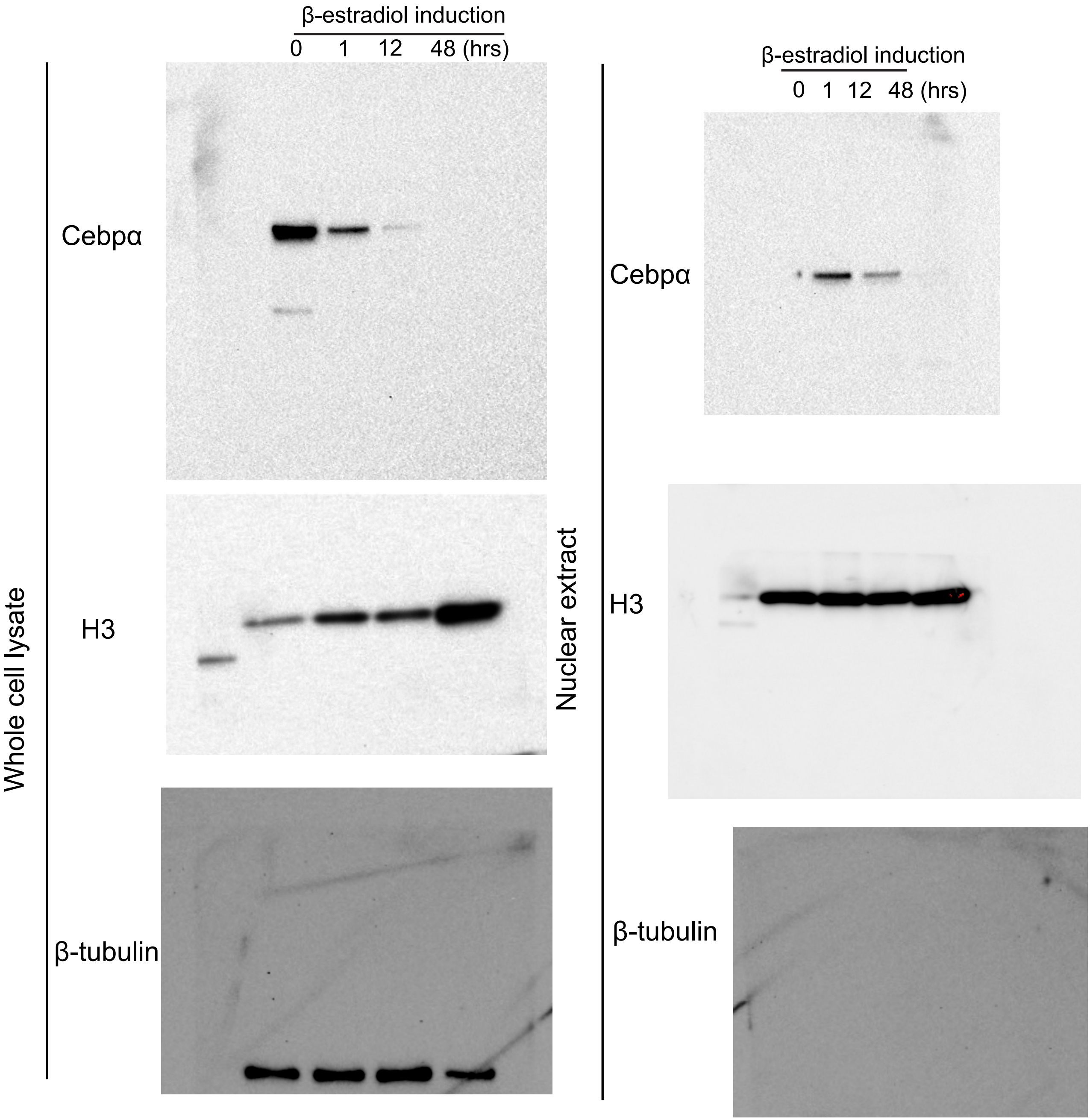

Fig. S8
